# Quality of Life Determinants in Patients with Metastatic Prostate Cancer: Insights from a Cross-Sectional Questionnaire-Based Study

**DOI:** 10.3390/curroncol31090366

**Published:** 2024-08-26

**Authors:** Chetanya Mittal, Hardik Gupta, Chitrakshi Nagpal, Ranjit K. Sahoo, Aparna Sharma, Bharat B. Gangadharaiah, Ghazal Tansir, Sridhar Panaiyadiyan, Shamim A. Shamim, Seema Kaushal, Chandan J. Das, Kunhi P. Haresh, Amlesh Seth, Brusabhanu Nayak, Atul Batra

**Affiliations:** 1 All India Institute of Medical Sciences, New Delhi 110029, India; chetanyamittal35@aiims.edu (C.M.); hardikg2001@aiims.edu (H.G.); 2 Department of Medical Oncology, Dr. B.R.A. Institute Rotary Cancer Hospital, All India Institute of Medical Sciences, New Delhi 110029, India; chitrakshinagpal11.jan20@aiims.edu (C.N.); drranjitmd@aiims.edu (R.K.S.); bharathbg01.jan21@aiims.edu (B.B.G.); ghzl_complique@yahoo.com (G.T.); 3 Department of Medical Oncology, National Cancer Institute, All India Institute of Medical Sciences, New Delhi 110029, India; aparnasharma@aiims.edu; 4 Department of Urology, National Cancer Institute, All India Institute of Medical Sciences, New Delhi 110029, India; sridharsoul@gmail.com; 5 Department of Nuclear Medicine, All India Institute of Medical Sciences, New Delhi 110029, India; sashamim2002@aiims.edu; 6 Department of Pathology, All India Institute of Medical Sciences, New Delhi 110029, India; seema.dr@aiims.edu; 7 Department of Radiodiagnosis, All India Institute of Medical Sciences, New Delhi 110029, India; chandan.das@aiims.edu; 8 Department of Radiation Oncology, Dr. B.R.A. Institute Rotary Cancer Hospital, All India Institute of Medical Sciences, New Delhi 110029, India; drkpharesh@gmail.com; 9 Department of Urology, All India Institute of Medical Sciences, New Delhi 110029, India; amlesh.seth@aiims.edu (A.S.); brusabhanu@aiims.edu (B.N.)

**Keywords:** prostate cancer, quality of life, health-related quality of life

## Abstract

Introduction: Prostate cancer is one of the most prevalent malignancies affecting men globally, with a significant impact on health-related quality of life (HRQOL). With the recent therapeutic advancements and improvements in survival, there is a need to understand the determinants of HRQOL in metastatic prostate cancer patients to optimize treatment strategies for quality of life as the number of survivors increases. The aim of this study was to identify clinical variables that affect HRQOL and its domains in patients with metastatic prostate cancer. Methods: We conducted a cross-sectional questionnaire-based study in patients diagnosed with metastatic prostate cancer at a tertiary cancer center in India. Baseline clinical features, treatment details, and completed Functional Assessment of Cancer Therapy—Prostate (FACT-P), composed of FACT-general (FACT-G) and prostate cancer-specific concerns subscale (PCS) and FACT-P Trial Outcome Index (FACT-P TOI) questionnaires, were collected. The mean total, as well as individual domain scores, were calculated. Additionally, these were stratified by the current treatment being received by patients. Linear regression was used to identify independent factors affecting HRQOL in these patients. Results: Of the 106 enrolled patients, 84 completed the FACT-P questionnaire and were included in the analysis. The median age was 66 years, and at the time of assessment, 3 patients (3.6%) were receiving androgen deprivation therapy only, 53 patients (63.1%) were on ADT + androgen receptor-targeted agents (ARTAs), and 18 patients (21.4%) patients received ADT + chemotherapy. The mean (±standard deviation) of the FACT-P TOI score was 70.33 (±15.16); the PCS subscale was the most affected, followed by functional well-being. Patients on chemotherapy scored significantly higher on PCS, but the composite scores were not significantly different. Univariable regression identified obesity (body mass index > 25 kg/m^2^) and duration of first-line treatment as significant predictors of better HRQOL; however, obesity was the only independent predictor in multivariable analysis (β = 8.2; 95% confidence interval, 1.2 to 15.0; *p* = 0.022). Obesity also independently predicted a better FACT-P and its physical well-being domain score and PCS. Conclusion: Prostate cancer patients experience impaired QoL, especially in the prostate cancer-specific and functional well-being domains. Lower BMI is an independent predictor of poor QoL, and this requires efforts to assess the impact of strategies to manage the nutritional status of patients with metastatic disease on QoL outcomes.

## 1. Introduction

Prostate cancer is the second most common malignancy in men worldwide [1]. The prognosis of prostate cancer has improved significantly due to recent advancements in therapies [2]. With an increasing number of patients living with metastatic prostate cancer, efforts are needed to understand the impact of the disease and its treatment on various dimensions of quality of life [3].

Health-related QOL (HRQOL) is a multidimensional assessment of how disease and its treatment affect a patient’s overall function and well-being [4]. Several validated HRQOL assessment tools are available for prostate cancer, of which the most common is the Functional Assessment of Chronic Illness Therapy-Prostate (FACT-P) questionnaire [5].

The FACT-P scale is a combination of the FACT-General (FACT-G) scale with four dimensions: physical well-being (PWB), social/family well-being (SWB), emotional well-being (EWB), and functional well-being (FWB), along with a 12-item prostate cancer-specific concerns subscale (PCS). It is a validated assessment tool for HRQOL with high content validity and internal consistency and is thus extensively used for QOL assessment in prostate cancer [6]. Higher scores indicate a better quality of life, and a change of 6 to 10 points is considered clinically meaningful [7,8]. Previous studies have shown mean baseline FACT-P scores (scored from 0 to 156) in patients with locally advanced/metastatic prostate cancer varying between 87.73 ± 19.88 and 116.62 ± 18.13 in the Indian and Western populations, respectively, suggesting that Indian patients with prostate cancer experience worse HRQOL than Western populations [9,10].

The prognostic association of baseline patient-reported HRQOL using the FACT-P scale has been extensively studied in localized prostate cancer [11]. An exploratory analysis of the CHAARTED trial revealed that low BMI is associated with significantly poor baseline HRQOL in patients with metastatic castration-sensitive prostate cancer, likely related to cancer-induced cachexia [12]. Apart from this, there have been very limited efforts to identify the status of patients with advanced disease, especially in the Indian setting, where HRQOL scores are comparatively lower than in the Western setting [9,10]. In this study, we aim to assess patient-reported HRQOL and identify prognostic factors that affect the quality of life in patients with metastatic prostate cancer [9,10].

## 2. Methodology

### 2.1. Study Design

The primary aim of this study was to identify the determinants of the HRQOL of patients with metastatic prostate cancer. We also sought to study the clinical variables affecting the various domains of HRQOL. It was a cross-sectional study conducted at the All India Institute of Medical Sciences (AIIMS), New Delhi, a tertiary cancer care center in India that caters to the populations of North India. Dr. B. R. A. Institute Rotary Cancer Hospital (IRCH), AIIMS, is the regional cancer center where approximately 15,000 patients with a new cancer diagnosis are treated annually [13].

**Figure 1:**
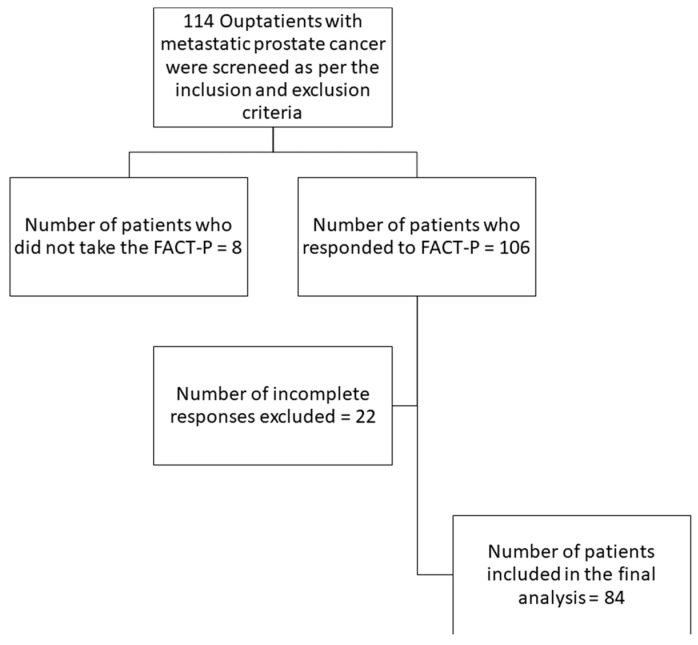
Enrollment of study participants and Health-Related Quality of Life Assessment. FACT-P: Functional Assessment of Cancer Therapy – Prostate.

This study was planned as a pilot study with a sample size of convenience screening and recruiting consecutive patients over 18 years of age with a histologically confirmed diagnosis of primary metastatic prostate cancer who were receiving systemic treatment at our center for a predetermined duration between September 2021 and June 2022. Patients with an expected life expectancy of <12 weeks, as determined by the treating oncologist, were excluded. All patients in this study had de novo metastatic prostate cancer, and none of them had been treated for local disease in the past (Figure 1). Demographic data were collected from all the participants, which included age, urban or rural residence (as reported by the patients), education status (categorized as illiterate—someone who cannot read and write [14]; the ones who were literate were categorized into those who have completed primary, middle, or high school or those who have a graduation or post-graduation degree), presence of comorbidities (comorbidities expected to be in a frequency of greater than 1% in our patient population were pre-specified and included in the case record form—diabetes mellitus, hypertension, hypothyroidism, CAD, CKD, stroke, and TIAs; the rest were classified as others), addictions, and accessibility to the hospital (calculated by measuring the distance from the patient’s PIN code to the PIN code of the hospital using Google Maps, 2021).

Clinical data collected included the duration of disease, site of metastasis, anthropometry (body mass index defined as per South-Asian cutoffs—underweight (<18.5 kg/m^2^), normal (18.5–22.9 kg/m^2^), overweight (23.0–24.9 kg/m^2^), and obese (≥25 kg/m^2^) [15], Eastern Cooperative Oncology Group (ECOG) performance status, and risk-stratification of metastatic disease (high-risk disease defined as ≥2 of the following: visceral metastasis, Gleason score ≥8, ≥3 bone lesions) [16], and burden of disease (high-volume disease defined as presence of visceral metastasis and/or ≥4 bone metastases, including at least one outside the vertebral bodies and pelvis) [17]. Laboratory values included serum prostate-specific antigen (PSA) at the time of assessment and the Gleason score of the prostate biopsy specimens. Treatment details collected included the type of androgen deprivation therapy received (ADT), the number of lines and types of treatment received, and their duration. The assessment of these baseline factors was stratified on the basis of the current therapy being received by the patient at the cross-sectional time point of assessment.

The institutional ethics committee approved the study (reference number IEC-646/03.09.2021, RP-23/2021). Written informed consent was obtained from all patients before their participation.

### 2.2. Assessment of HRQOL

We utilized the FACT-P measure to determine the HRQOL, which has been concluded to be the most appropriate patient-reported outcome measure for patients with metastatic prostate cancer by the PIONEER (Prostate Cancer DIagnOsis and TreatmeNt Enhancement through the Power of Big Data in EuRope) consortium [6]. All participants were assessed using this questionnaire at a single time point (in Hindi or English according to the patient’s comprehension) [18]. The license for the English and Hindi versions for clinical providers was obtained in the institution’s name from the Functional Assessment of Chronic Illness Therapy (FACIT) System Organization in the name of the institution vide agreement dated 9 August 2021.

Self-administration of the measure was the preferred mode of administering the questionnaire. Patients with difficulty comprehending the questions received assistance from our research staff, limited to explaining the literal meaning of the question in further, simpler terms without interfering with the responses. The FACT-P scale consists of the FACT-G and PCS subscales. The FACT-G (version 4) is a 27-item questionnaire comprising four dimensions (PWB, SWB, EWB, and FWB). Each domain has seven items and is scored out of 28, except EWB (6 items, scored out of 24) [7]. All items are answered on a Likert scale ranging from 0 to 4, with 0 representing “not at all” and 4 representing “very much.” The subscale comprises 12 items encompassing bowel and bladder function, sexual activity, and pain, and is scored out of 48. The combination of the scales provides a global HRQOL score and domain-specific scores [19]. A modified version of FACT-P, called the FACT-P trial outcome index (FACT-P TOI), includes only the physical and functional domain scores and the PCS subscale. It takes less time to fill, is more sensitive than the FACT-P score, and is thus extensively used as an end-point in clinical trials [20]. The FACT-P is scored from 0 to 156, the FACT-G from 0 to 108, and the FACT-P TOI from 0 to 104. A higher score is indicative of a better quality of life.

A response rate of at least 50% in each subscale and 80% overall was required to include incompletely filled questionnaires. If less than 50% of individual items were skipped, subscale scores were prorated using the average of the other answers in the scale. This imputation method is standard across The Functional Assessment of Chronic Illness Therapy (FACIT) measurement system [20]. Patients with less than 50% of the forms filled out were excluded from the study.

### 2.3. Statistics

Descriptive statistics were used to present demographics and baseline clinical parameters. Categorical data were described as percentages, and continuous variables were described as mean (± standard deviation). The means of the FACT-P, FACT-P TOI, and FACT-G scores were calculated as the composite scores and domain-wise subscores of the FACT-P tool and demonstrated as box and whiskers plots. Potential predictive factors associated with better or worse FACT-P TOI, FACT-P, and FACT-G scores were identified using linear regression analysis. Univariable linear regression analysis was performed on all the baseline demographic and clinical factors reported with the linear regression coefficient, beta, and its 95% confidence interval. Those with a *p*-value of <0.05 or previously studied prognostic factors were included in the multivariable analysis. The significant *p*-value used was specified as <0.05 a priori. Statistical analysis was performed using R version 4.3.2 (RStudio, R Foundation for Statistical Computing, Vienna, Austria).

## 3. Results

### 3.1. Baseline Characteristics

A total of 106 patients with metastatic prostate cancer were screened during the study period, and they consented to complete the questionnaire. A total of 84 patients who completed a FACT-P measure with an overall response rate of >80% were included in the final analysis. A total of 3 patients (3.6%) were receiving androgen deprivation therapy only; 53 patients (63.1%) were on ADT + androgen receptor-targeted agents (ARTAs)—90.6% abiraterone and 9.4% enzalutamide. A total of 18 patients (21.4%) received ADT + chemotherapy—docetaxel—94% and cabazitaxel—5.6%. The median age of the participants was 66 (59–71) years; one-third belonged to a rural area (35%), and around 10% were illiterate. About half of the patients (48%) had one or more comorbid medical conditions (35% hypertension and 15% diabetes mellitus), and 46% of patients were obese. The median duration from diagnosis of metastatic prostate cancer to the assessment of HRQOL was 11 months (IQR, 4–38), with a higher median duration—25 months in chemotherapy patients versus 10 months in those receiving ADT/ADT + ARTA therapy, 65% had high-risk disease, and 62% of them had high-volume disease. Almost equal numbers had castration-sensitive and resistant diseases, respectively. Most of our patients had received androgen deprivation therapy; 69% underwent bilateral orchiectomy; and the remaining received medical castration. Forty-one percent of our patients received more than one line of treatment. Abiraterone was the choice of first-line therapy in 72% of patients, with others having received docetaxel (23%), enzalutamide (2.7%), and fosfestrol (2.7%). The median duration of first-line treatment was one year (IQR, 5–21 months). As of 31 December 2023, 45 (54%) patients in our cohort were alive (Table 1).

### 3.2. Quality of Life

**Figure 2:**
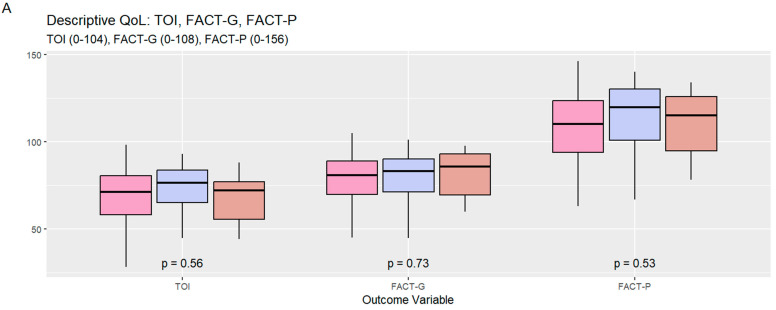

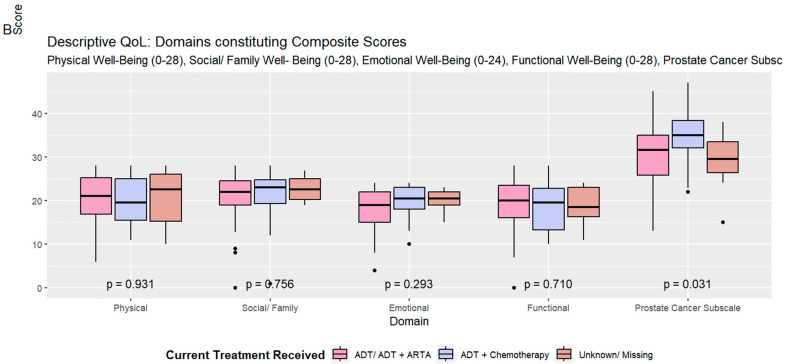
Composite outcome scores (**A**) and domain-wise score constituting FACT-P (**B**) stratified by the current treatment received; FACT-P: Functional Assessment of Cancer Therapy-Prostate; FACT-G: Functional Assessment of Cancer Therapy-General; TOI: Trial Outcome Index; ADT: androgen deprivation therapy; ARTA: androgen receptor-targeted agent.

The mean composite FACT-P, FACT-P TOI, and FACT-G scores and their respective standard deviations (SD) were 110.27 (20.18), 70.33 (15.16), and 79.25 (15.02), respectively. The domain-wise mean scores and SD for the subscales of physical well-being (PWB), social/family well-being (SWB), emotional well-being (EWB), functional well-being (FWB), and prostate cancer-specific subscale (PCS) were 20.41 (5.69), 21.24 (5.46), 18.70 (4.39), 18.90 (5.60), and 31.02 (7.10), respectively. Patients on chemotherapy had a significantly higher PCS (*p* = 0.03); however, the distributions were not significantly different for the composite scores across these subgroups (Figure 2 and Table 2).

### 3.3. Factors Affecting HRQoL

In univariable regression analysis, the FACT-P TOI was significantly better in patients with a longer duration of first-line treatment received (β = 0.28; 95% confidence interval [CI], 0.04 to 0.52; *p* = 0.02) and those with a higher body mass index (BMI) (β = 6.80; 95% CI, 0.30 to 13.0; *p* = 0.04) (Table 3). On multivariable analysis, BMI > 25 was the only factor associated with a better QOL (β = 8.20; 95% CI, 1.20 to 15.0; *p* = 0.02). Additionally, obesity (BMI > 25) was also an independent predictor of a higher FACT-P score (β = 11.0; 95% CI, 1.60 to 21.0; *p* = 0.02). BMI > 25 also correlated positively with the composite FACT-G score (β = 6.50; 95% CI, 0.05 to 13.0; *p* = 0.05), while higher PSA levels were negatively affecting the total FACT-G score (β = −0.01; 95% CI, −0.02 to 0.00; *p* = 0.03). On multivariable analysis, serum PSA levels independently predicted a worse FACT-G score, and obesity had a trend towards significance (β = 7.20; 95% CI, −0.10 to 14.0; *p* = 0.054) on multivariable analysis (Table 4).

We also performed a domain-wise analysis with similar predictor variables. Obesity, duration of first-line treatment, and the current treatment received were significant predictors for the PCS subscale on univariable analysis; obesity and current treatment received were independently predictive on multivariable analysis (β = 4.20; 95% CI, 0.60 to 7.80; *p* = 0.02; β = 7.9; 95% CI, 3.0 to 13.0; *p* = 0.02). In the multivariable analysis, a higher ECOG PS score was associated with worse physical QOL (β = −3.3; 95% CI −6.20 to −0.50; *p* = 0.02), and obesity was significantly associated with a higher physical well-being domain score (β = 2.8; 95% CI, 0.1 to 5.5; *p* = 0.04). Higher PSA was found to impact emotional well-being (β (per 10 units change in PSA) = −0.029; 95% CI, −0.053 to −0.005; *p* = 0.02), and this association was consistent after adjusting for the other variables in multivariable analysis (β (per 10 units change in PSA) = −0.03; 95% CI, −0.06 to 0.00; *p* = 0.03) (Tables S1 and S2).

## 4. Discussion

We present cross-sectional HRQOL data from 84 patients with metastatic prostate cancer. We found that Indian patients with metastatic prostate cancer had a mean FACT-P QOL of 110.27 (SD ± 20.18). Weighing individual domains with the maximum scores, the worst affected domains included the prostate-specific subscale (including bowel and bladder function, sexual activity, and pain), followed by functional well-being. Further, patients with a higher BMI (>25 kg/m^2^) had a better quality of life when adjusted for other baseline factors, including the prostate-specific subscale. There was no significant difference between the HRQOL outcomes between patients receiving ARTAs or chemotherapy at the time of assessment in our study cohort, except for the prostate-cancer-specific concerns subscale.

Indian population reference values are not available for the FACT-P questionnaire. However, compared with the United States reference values for the FACT-G scale, physical well-being is the most affected out of all domains in patients with metastatic prostate cancer, as shown in Figure S1 [21]. Surprisingly, our patients had higher scores for SWB [21.24 (5.6) versus 17.2 (6.9)], which may be due to the presence of a more cohesive social and family structure in the Indian setting, where the elderly often reside with their children in a joint family and are thus a source of social support [22]. Our outcome parameters were in close agreement with the baseline parameters reported by a Canadian study [23], with a FACT-P score of 111.3 (19.56) and similar means across domains. A study enrolling 280 patients from Europe, Australia, and North America reported a total FACT-P score of 105.1 ± 22.5 versus 110.27 ± 20.18 in our study [24]. This study enrolled patients with metastatic hormone-resistant prostate cancer, and this may explain the similar or lower HRQOL scores across FACT-G, FACT-P, and all its domains, as our study has almost an equal proportion of patients with CSPC and CRPC. Additionally, our patients had a better quality of life when compared with other metastatic solid organ tumors (lung, breast, cervical, and oral malignancies mainly), as depicted by the FACT-G scale [25].

We found that BMI was an independent predictor of quality of life scores. A lower BMI is the strongest predictor of a poor quality of life. The relationship between baseline BMI and HRQOL was explored in the CHAARTED study, and it was hypothesized that patients in the low BMI group had a poorer QOL because of the higher disease burden in these patients [12]. However, we found no significant difference in the disease burden between the two groups in our cohorts. The low BMI in our patients likely represents cancer-induced cachexia or weight loss due to disease activity. This causes significant impairment and a worse perception of the disease effect [26]. Patients with a high BMI have fat reserves, which may prevent cancer cachexia from manifesting [27]. Additionally, obese men are likely to have a higher estrogen level in their bodies due to peripheral conversion, which might be responsible for inhibiting tumor growth [27]. This relationship is similar to other cancers, such as breast cancer, where significant weight loss after diagnosis is an independent prognostic indicator [28]. However, it is noteworthy that obesity is associated with a higher recurrence rate, and the association is well-established in estrogen receptor-positive breast cancer [29]. Notably, sarcopenia and obesity may co-exist in men with androgen deprivation [30]. However, high BMI has previously been shown to have better outcomes, irrespective of the presence of sarcopenia [30]. However, it is relevant to note that due to the heterogeneous nature of our study population, this result may also reflect post-chemotherapy weight loss in the subset of patients on ADT + chemotherapy experiencing a worse HRQOL due to the effects of chemotherapy. We adjusted for this in the multivariable regression and noted worsening HRQOL with ADT + Docetaxel as 1st line therapy compared to ADT + Abiraterone (β = −14; 95% CI, −26 to −1.8), but the association was not significant (*p* = 0.15). 

In addition to higher BMI, we found that serum PSA levels significantly predicted the FACT-G score but not the FACT-P scores. This reflects the variance of various composite scores for different predictor variables. Patients with a higher PSA had worse composite general scores. This is likely due to the higher disease burden, as is shown in the secondary analysis of the ALSYMPCA trial, where worsening of PSA overtime is associated with worse FACT-G scores [31]. The validation study for FACT-P also reported the statistically significant sensitivity of FACT-G to a change in PSA levels. However, they reported no statistically significant domain-wise changes, and we observed significant improvement in emotional well-being in patients with lower PSA levels [7]. This is expected as PSA anxiety is a prominent symptom for prostate cancer patients, with a prevalence of around 22%, and has been associated with poorer quality of life [32]. It is also interesting to note that while serum PSA levels are significantly associated with the FACT-G score, variables such as tumor load or type of metastasis do not seem to have an effect. Serum PSA levels are taken at the time of assessment; therefore, they represent the current status more accurately, and thus, they correlate better with HRQOL. In alia manu, the imaging studies (PET-CT) represent an earlier disease burden as they would have been conducted at an earlier time point and do not have a significant correlation with HRQOL.

Poorer ECOG performance status was associated with worse physical well-being, but not with the composite FACT-P score. ECOG is more directly representative of the physical status of the patient, minimizing its contribution to the composite score. This is somewhat in agreement with the FACT-P validation study, where, in addition to physical well-being, functional well-being, and the prostate cancer subscale, TOI and FACT-P had statistically significant sensitivity to changes in ECOG PS [7]. Performance status has previously been linked with worse physical symptoms using the National Comprehensive Cancer Network/Functional Assessment of Cancer Therapy—Prostate Symptom Index-Physical Scale (NFPSI-P) at baseline and after treatment [31]. ECOG PS has previously been identified as a strong prognostic factor in patients with metastatic prostate cancer [33]. This association of ECOG performance status with physical well-being and survival has also been shown in patients with metastatic lung cancer [34].

The striking absence of a lack of significant differences in HRQOL between the patients receiving chemotherapy and ARTAs may be hypothesized to reflect the advanced nature of metastatic prostate cancer, where the disease has more impact on the quality of life as opposed to the adverse effect profile of the patient’s treatment. This may also reflect better disease control with chemotherapy. This is supported by the observation that patients on chemotherapy had a significantly better score on the prostate-cancer-specific concerns subscale (β = 7.9; 95% CI, 3.0 to 13.0; *p* = 0.02). Our results are in agreement with the results of the TAX 327 study, which suggests that docetaxel, despite its toxicity, has efficacious palliative effects as assessed by the FACT-P measure, with the greatest benefit for the PCS subscale [35].

The limitations of our study include the cross-sectional nature of a relatively small sample size, which represents only a screenshot of the data with no follow-up data after therapy. Additionally, the lack of population reference for the Indian subcontinent makes it difficult to accurately understand the magnitude of the deterioration of quality of life. A domain-wise analysis is difficult with FACT-P, where the contribution of domains depends on the number of items; weighted domain-wise subscores to calculate an adjusted FACT-P approach still remain to be validated [36]. The various indices have slight variations in the sensitivities to multiple dependent variables, and there is a possibility of type 1 statistical errors. Moreover, data and evidence are lacking as to what extent HRQOL can be improved by manipulation of the cachectic state by nutritional therapies or otherwise. Ours is one of the first studies in India to assess HRQOL in patients with prostate cancer and their demographic and cultural predictors individually.

## 5. Conclusions

The FACT-P measure is a useful patient-reported outcome measure assessing HRQOL in patients with metastatic prostate cancer in a tertiary care setting. However, there is a need to develop an independent population reference sample in India for FACT-G and FACT-P. We found that low BMI is an independent predictor of poor QoL. Thus, efforts are required to develop strategies to manage the nutritional status of patients with metastatic disease and to prospectively assess such interventions and their impact on HRQOL. Doublet therapy was the contemporary standard of care during the period of the study. Based on the change in institutional protocols, patients at our center have now been receiving triplet therapy, and it will also be of interest to us to study HRQOL in these patients. Future prospective studies that include a longitudinal follow-up of patients will provide more insight into how HRQOL changes as the disease progresses and with therapy and will help expand the use of the FACT-P questionnaire in our setting.

## Figures and Tables

**Table 1:** Baseline Characteristics; ^1^M: N Missing (% Missing), IQR, interquartile range; ADT, androgen deprivation therapy; TIAs, transient ischemic attacks; AIDS, acquired immunodeficiency syndrome; Ca Bladder, carcinoma bladder; BMI, body mass index; ECOG, Eastern Cooperative Oncology Group; PSA, prostate-specific antigen; ADT, androgen deprivation therapy; ^¶^ High-volume disease was defined as per the protocol of the CHAARTED trial by Sweeney et al., 2015 [17].

Characteristic	^1^M	Overall, N = 84
**Age, Median (IQR)**		66 (59–71)
**Area, n (%)**		
*Rural*		29 (35)
*Urban*		55 (65)
**Education, n (%)**		
*Illiterate*		8 (9.5)
*Schooling*		47 (56)
*Graduate and above*		29 (35)
**Duration of Disease (Months), Median (IQR)**	10 (12)	11 (4–38)
**Comorbidities, n (%)**		
*Diabetes Mellitus, n (%)*		15 (18)
*Hypertension, n (%)*		29 (35)
*Hypothyroidism, n (%)*		2 (2.4)
*Coronary Artery Disease, n (%)*		5 (6.0)
*Chronic Kidney Disease, n (%)*		1 (1.2)
*Stroke and TIAs, n (%)*		2 (2.4)
*Other (AIDS, Hepatitis B, Ca Bladder, Hernia), n (%)*		4 (4.8)
**Number of Comorbidities, n (%)**		
*None*		44 (52)
*1 Comorbidity*		29 (35)
*>1 Comorbidity*		11 (13)
**Tobacco (Smokeless), n (%)**		49 (58)
**Smoking, n (%)**		30 (36)
**Alcohol, n (%)**		25 (30)
**Type of Metastasis, n (%)**	5 (6.0)	
*Bony metastasis*		22 (28)
*Visceral metastasis*		9 (11)
*Bony and Visceral metastasis*		48 (61)
**Accessibility to AIIMS (kilometers), Median (IQR)**	2 (2.4)	70 (21–400)
**BMI, n (%)**		
*Underweight (< 18.5)*		6 (7.1)
*Normal (18.5–22.9)*		25 (30)
*Overweight (23–24.9)*		14 (17)
*Obese (> 25)*		39 (46)
**ECOG Status, n (%)**	4 (4.8)	
*ECOG (0–1)*		47 (59)
*ECOG (2–3)*		33 (41)
**PSA (ng/mL), Median (IQR)**	3 (3.6)	7 (1–71)
**Gleason Score, n (%)**	6 (7.1)	
*6 and 7, Low and Medium Risk*		19 (24)
*8, High Risk*		23 (29)
*9 and 10, High Risk*		36 (46)
**Castration Sensitivity, n (%)**	5 (6.0)	
*Castration-Sensitive, CSPC*		39 (49)
*Castration-Resistant, CRPC*		40 (51)
**Type of ADT, n (%)**	6 (7.1)	
*Medical*		24 (31)
*Surgical*		54 (69)
**Risk-Stratification, n (%)**	5 (6.0)	
*Low-risk*		28 (35)
*High-risk*		51 (65)
**Burden of Disease, n (%)** ^¶^	5 (6.0)	
*Low-volume*		30 (38)
*High-volume*		49 (62)
**Number of Treatment Lines, n (%)**	5 (6.0)	
*1 Line of Treatment*		47 (59)
*>1 Lines of Treatment*		32 (41)
**1st Line Treatment Received, n (%)**	10 (12)	
*ADT + Abiraterone*		53 (72)
*ADT + Docetaxel*		17 (23)
*ADT + Enzalutamide*		2 (2.7)
*ADT + Fosfosterol*		2 (2.7)
**Treatment at the Time of Assessment, n (%)**	10 (12)	
*ADT + Abiraterone*		51 (69)
*ADT + Docetaxel*		17 (23)
*ADT + Enzalutamide*		5 (6.8)
*ADT + Cabazitaxel*		1 (1.4)
**Duration of 1st Line Treatment (Months), Median (IQR)**	13 (15)	12 (5–21)
**Current Status, n (%)**		
*Alive*		45 (54)
*Dead*		39 (46)

**Table 2:** Domain-wise HRQOL; ADT, androgen deprivation therapy; ARTA, androgen receptor-targeted agent.

Characteristic	Overall, N = 84	ADT + ARTA, N = 56	ADT + Chemotherapy, N = 18	Unknown/Missing, N = 10	*p*-Value ^1^
**Physical Well-Being (0–28)**					0.93
*Mean (SD)*	20.41 (5.69)	20.37 (5.64)	20.31 (5.75)	20.82 (6.48)	
**Social/Family Well-Being (0–28)**					0.76
*Mean (SD)*	21.24 (5.46)	20.98 (5.46)	21.29 (6.63)	22.60 (2.74)	
**Emotional Well-Being (0–24)**					0.29
*Mean (SD)*	18.70 (4.39)	18.10 (4.70)	19.78 (3.90)	20.10 (2.64)	
**Functional Well-Being (0–28)**					0.71
*Mean (SD)*	18.90 (5.60)	19.14 (5.77)	18.43 (5.69)	18.40 (4.88)	
**Prostate Cancer Subscale (0–48)**					**0.031**
*Mean (SD)*	31.02 (7.10)	30.15 (7.09)	34.82 (6.36)	29.06 (6.57)	
**FACT-P TOI (Trial Outcome Index) (0–104)**					0.56
*Mean (SD)*	70.33 (15.16)	69.66 (15.49)	73.56 (14.33)	68.28 (15.43)	
**FACT-G (General Cancer) (0–108)**					0.73
*Mean (SD)*	79.25 (15.02)	78.59 (15.27)	79.81 (15.23)	81.92 (14.33)	
**FACT-P (Prostate Cancer) (0–156)**					0.53
*Mean (SD)*	110.27 (20.18)	108.74 (20.46)	114.62 (20.02)	110.98 (19.63)	

^1^ Kruskal–Wallis rank sum test. Bold values denote statistical significance at the *p* < 0.05 level.

**Table 3:** Univariable Regression Analysis; FACT-P: Functional Assessment of Cancer Therapy—Prostate; FACT-G: Functional Assessment of Cancer Therapy—General; TOI: Trial Outcome Index; ADT, androgen deprivation therapy; ARTA, androgen receptor-targeted agent; ECOG, Eastern Cooperative Oncology Group; PSA, prostate-specific antigen; BMI, body mass index.

Characteristic	TOI		FACT-G		FACT-P	
	Beta (95% CI) ^1^	*p*-Value	Beta (95% CI) ^1^	*p*-Value	Beta (95% CI) ^1^	*p*-Value
**Age**	−0.14 (−0.51 to 0.23)	0.44	−0.05 (−0.42 to 0.32)	0.78	−0.17 (−0.67 to 0.32)	0.49
**Area**		0.72		0.95		0.82
*Rural*	—		—		—	
*Urban*	1.3 (−5.7 to 8.2)		−0.21 (−7.1 to 6.7)		−1.0 (−10 to 8.2)	
**Accessibility to AIIMS with respect to Median Distance**		0.13		0.92		0.60
*Lesser/Equal*	—		—		—	
*Greater*	−5.1 (−12 to 1.6)		−0.33 (−7.0 to 6.3)		−2.3 (−11 to 6.6)	
**Education**		0.97		0.67		0.93
*Illiterate*	—		—		—	
*Schooling*	0.36 (−11 to 12)		2.6 (−8.9 to 14)		1.2 (−14 to 17)	
*Graduate and above*	1.2 (−11 to 13)		4.9 (−7.1 to 17)		2.6 (−14 to 19)	
**Duration of Disease with respect to Median Duration**		0.88		0.63		0.61
*Lesser/Equal*	—		—		—	
*Greater*	0.55 (−6.6 to 7.8)		1.7 (−5.4 to 8.9)		2.4 (−7.1 to 12)	
**Number of Comorbidities**		0.63		0.81		0.87
*None*	—		—		—	
*1 Comorbidity*	0.65 (−6.6 to 7.9)		2.3 (−4.9 to 9.5)		1.2 (−8.5 to 11)	
*>1 Comorbidity*	−4.4 (−15 to 5.8)		0.33 (−9.8 to 10)		−2.7 (−16 to 11)	
**Tobacco (Smokeless)**	−2.6 (−9.3 to 4.1)	0.44	−0.68 (−7.3 to 6.0)	0.84	−0.66 (−9.6 to 8.3)	0.88
**Alcohol**	0.54 (−6.7 to 7.8)	0.88	2.4 (−4.7 to 9.6)	0.50	0.93 (−8.7 to 11)	0.85
**Type of Metastasis**		0.23		0.48		0.32
*Bony metastasis*	—		—		—	
*Visceral metastasis*	−10 (−22 to 1.5)		−7.2 (−19 to 4.7)		−12 (−28 to 3.7)	
*Bony and Visceral metastasis*	−3.2 (−11 to 4.4)		−1.4 (−9.1 to 6.3)		−3.7 (−14 to 6.5)	
**Obese: BMI > 25**	6.8 (0.31 to 13)	**0.040**	6.5 (0.05 to 13)	**0.048**	9.6 (1.0 to 18)	**0.028**
**ECOG**		0.072		0.44		0.36
*ECOG (0–1)*	—		—		—	
*ECOG (2–3)*	−6.2 (−13 to 0.57)		−2.7 (−9.5 to 4.1)		−4.2 (−13 to 4.9)	
**PSA**	−0.01 (−0.01 to 0.00)	0.16	−0.01 (−0.02 to 0.00)	**0.029**	−0.01 (−0.02 to 0.00)	0.062
**Gleason Score Category**		0.96		0.44		0.79
*6 and 7, Low and Medium Risk*	—		—		—	
*8, High Risk*	−0.36 (−9.8 to 9.1)		−4.0 (−13 to 5.3)		−2.9 (−16 to 9.8)	
*9 and 10, High Risk*	0.75 (−7.9 to 9.4)		1.1 (−7.5 to 9.7)		0.78 (−11 to 12)	
**Castration Sensitivity**		0.15		0.40		0.30
*Castration-Sensitive, CSPC*	—		—		—	
*Castration-Resistant, CRPC*	−5.0 (−12 to 1.9)		−2.9 (−9.7 to 3.9)		−4.9 (−14 to 4.3)	
**Type of ADT Received**		0.53		0.67		0.85
*Medical*	—		—		—	
*Surgical*	−2.4 (−10 to 5.2)		1.6 (−5.9 to 9.1)		0.95 (−9.1 to 11)	
**Burden of Disease**		0.37		0.24		0.24
*Low-volume*	—		—		—	
*High-volume*	−3.2 (−10 to 3.9)		−4.2 (−11 to 2.8)		−5.6 (−15 to 3.9)	
**Number of Treatment Lines Received**		0.60		0.72		0.73
*1 Line of Treatment*	—		—		—	
*>1 Lines of Treatment*	−1.9 (−8.9 to 5.2)		−1.3 (−8.2 to 5.7)		−1.7 (−11 to 7.8)	
**1st Line Treatment Received**		0.053		0.055		0.065
*Abiraterone*	—		—		—	
*Docetaxel*	−9.7 (−18 to −1.6)		−9.8 (−18 to −1.7)		−12 (−23 to −1.1)	
*Enzalutamide*	5.5 (−16 to 27)		3.5 (−18 to 25)		7.3 (−21 to 36)	
*Fosfosterol*	12 (−9.0 to 33)		12 (−8.8 to 33)		18 (−10 to 47)	
**Duration of First-Line Treatment Received**	0.28 (0.04 to 0.52)	**0.020**	0.19 (−0.06 to 0.43)	0.13	0.31 (−0.02 to 0.63)	0.063
**Current Treatment Received**		0.35		0.77		0.29
*ADT + ARTA*	—		—		—	
*ADT + Chemotherapy*	3.9 (−4.3 to 12)		1.2 (−7.0 to 9.5)		5.9 (−5.1 to 17)	

^1^ CI = Confidence Interval. Bold values denote statistical significance at the *p* < 0.05 level.

**Table 4:** Multivariable Regression Analysis; ECOG, Eastern Cooperative Oncology Group; PSA, prostate-specific antigen; BMI, body mass index.

Characteristic	TOI		FACT-G		FACT-P	
	Beta (95% CI) ^1^	*p*-Value	Beta (95% CI) ^1^	*p*-Value	Beta (95% CI) ^1^	*p*-Value
**Age**	−0.28 (−0.71 to 0.15)	0.20	−0.17 (−0.62 to 0.28)	0.45	−0.40 (−0.99 to 0.20)	0.19
**Number of Comorbidities**		0.35		0.96		0.69
*None*	—		—		—	
*1 Comorbidity*	−3.0 (−11 to 5.3)		−0.54 (−9.1 to 8.1)		−2.2 (−14 to 9.2)	
*>1 Comorbidity*	−7.9 (−19 to 3.5)		−1.7 (−14 to 10)		−6.5 (−22 to 9.1)	
**Type of Metastasis**		0.31		0.60		0.55
*Bony metastasis*	—		—		—	
*Visceral metastasis*	−10 (−23 to 3.1)		−5.8 (−19 to 7.8)		−9.5 (−27 to 8.4)	
*Bony and Visceral metastasis*	−2.0 (−10 to 6.1)		0.44 (−8.0 to 8.8)		−1.3 (−12 to 9.8)	
**Obese: BMI > 25**	8.2 (1.2 to 15)	**0.022**	7.2 (−0.12 to 14)	0.054	11 (1.6 to 21)	**0.023**
**ECOG**	−4.9 (−12 to 2.6)	0.19	−2.7 (−10 to 5.1)	0.49	−3.1 (−13 to 7.2)	0.55
**PSA**	−0.06 (−0.14 to 0.03)	0.19	−0.08 (−0.17 to 0.00)	0.061	−0.09 (−0.21 to 0.03)	0.12
**1st Line Treatment Received**		0.11		0.13		0.15
*Abiraterone*	—		—		—	
*Docetaxel*	−11 (−20 to −1.9)		−11 (−20 to −1.8)		−14 (−26 to −1.8)	
*Enzalutamide*	4.9 (−26 to 35)		2.8 (−29 to 35)		8.4 (−34 to 50)	
*Fosfosterol*	−3.7 (−28 to 20)		4.7 (−20 to 30)		1.0 (−32 to 34)	
**Duration of First-Line Treatment Received**	0.14 (−0.16 to 0.44)	0.35	0.04 (−0.27 to 0.35)	0.81	0.12 (−0.29 to 0.53)	0.56

^1^ CI = Confidence Interval. Bold values denote statistical significance at the *p* < 0.05 level.

## Data Availability

The datasets used and/or analyzed during the current study are available from the corresponding author upon reasonable request.

## Supplementary Materials

The following supporting information can be downloaded at: https://www.mdpi.com/article/10.3390/curroncol31090366/s1, Figure S1: Domain-wise scores constituting FACT-P compared with United States population reference values.; Table S1: Domain-wise univariable regression analysis; Table S2: Domain-wise multivariable regression analysis.
