# Supplementary material for: Quality of Life Determinants in Patients with Metastatic Prostate Cancer: Insights from a Cross-Sectional Questionnaire-Based Study
Source: Curr Oncol. 2024 Aug 26;31(9):4940–54. doi: 10.3390/curroncol31090366 (PMC11430678; doi:10.3390/curroncol31090366)
Supplement: Supplementary file 1 [file curroncol-31-00366-s001.zip › Supplementary/Table S1.pdf]

Characteristic	Physical			Social			Emotional			Functional			Prostate Cancer Subscale		
	N	Beta (95% CI)[1]	p-value	N	Beta (95% CI)[1]	p-value	N	Beta (95% CI)[1]	p-value	N	Beta (95% CI)[1]	p-value	N	Beta (95% CI)[1]	p-value
**Age**	84	0.02 (-0.12 to 0.16)	0.79	84	-0.06 (-0.19 to 0.08)	0.39	84	0.029 (-0.079 to 0.136)	0.60	84	-0.04 (-0.18 to 0.10)	0.56	84	-0.12 (-0.29 to 0.05)	0.16
**Area**	84		0.24	84		0.34	84		0.28	84		0.68	84		0.61
Rural		—			—			—			—			—	
Urban		1.6 (-1.0 to 4.1)			-1.2 (-3.7 to 1.3)			-1.094 (-3.096 to 0.908)			0.53 (-2.0 to 3.1)			-0.83 (-4.1 to 2.4)	
**Accessibility to AIIMS with respect to Median Distance**	82		0.10	82		0.10	82		0.45	82		0.43	82		0.21
Lesser/ Equal		—			—			—			—			—	
Greater		-2.1 (-4.5 to 0.42)			2.0 (-0.42 to 4.4)			0.741 (-1.213 to 2.696)			-0.98 (-3.5 to 1.5)			-2.0 (-5.1 to 1.1)	
**Education**	84		0.83	84		0.38	84		0.67	84		0.12	84		0.71
Illiterate		—			—			—			—			—	
Schooling		-1.3 (-5.7 to 3.1)			1.2 (-2.9 to 5.4)			-0.439 (-3.804 to 2.926)			3.1 (-1.1 to 7.3)			-1.4 (-6.8 to 4.1)	
Graduate and above		-1.0 (-5.6 to 3.6)			2.7 (-1.7 to 7.0)			-1.251 (-4.765 to 2.263)			4.5 (0.11 to 8.9)			-2.3 (-7.9 to 3.4)	
**Duration of Disease with respect to Median Duration**	73		0.84	73		0.44	73		0.43	73		0.92	73		0.68
Lesser/ Equal		—			—			—			—			—	
Greater		-0.28 (-3.0 to 2.4)			1.0 (-1.6 to 3.7)			0.860 (-1.290 to 3.009)			0.14 (-2.5 to 2.8)			0.70 (-2.6 to 4.0)	
**Number of Comorbidities**	84		0.89	84		0.78	84		0.94	84		0.30	84		0.45
None		—			—			—			—			—	

Characteristic	Physical			Social			Emotional			Functional			Prostate Cancer Subscale		
	**N**	**Beta (95% CI)[1]**	**p-value**	**N**	**Beta (95% CI)[1]**	**p-value**	**N**	**Beta (95% CI)[1]**	**p-value**	**N**	**Beta (95% CI)[1]**	**p-value**	**N**	**Beta (95% CI)[1]**	**p-value**
1 Comorbidity		-0.14 (-2.9 to 2.6)			0.52 (-2.1 to 3.1)			0.042 (-2.072 to 2.155)			1.9 (-0.78 to 4.5)			-1.1 (-4.5 to 2.3)	
>1 Comorbidity		-0.94 (-4.8 to 2.9)			1.2 (-2.5 to 4.9)			0.532 (-2.447 to 3.510)			-0.50 (-4.2 to 3.2)			-3.0 (-7.8 to 1.8)	
**Tobacco (Smokeless)**	84	-1.3 (-3.8 to 1.2)	0.30	84	1.7 (-0.68 to 4.1)	0.16	84	0.236 (-1.708 to 2.180)	0.81	84	-1.3 (-3.8 to 1.2)	0.30	84	0.02 (-3.1 to 3.2)	0.99
**Alcohol**	84	1.4 (-1.3 to 4.1)	0.31	84	1.2 (-1.4 to 3.8)	0.37	84	-0.788 (-2.878 to 1.302)	0.46	84	0.65 (-2.0 to 3.3)	0.63	84	-1.5 (-4.9 to 1.9)	0.37
**Type of Metastasis**	79		0.50	79		0.94	79		0.39	79		0.20	79		0.18
Bony metastasis		—			—			—			—			—	
Visceral metastasis		-2.2 (-6.6 to 2.2)			0.54 (-3.9 to 5.0)			-2.389 (-5.916 to 1.138)			-3.1 (-7.5 to 1.3)			-4.9 (-10 to 0.60)	
Bony and Visceral metastasis		-1.5 (-4.3 to 1.4)			0.46 (-2.4 to 3.4)			-0.967 (-3.262 to 1.328)			0.56 (-2.3 to 3.4)			-2.3 (-5.9 to 1.3)	
**Obese: BMI >25**	84	2.1 (-0.30 to 4.6)	0.085	84	1.2 (-1.2 to 3.6)	0.32	84	1.646 (-0.242 to 3.534)	0.087	84	1.5 (-0.95 to 3.9)	0.23	84	3.2 (0.13 to 6.2)	**0.041**
**ECOG**	80		0.056	80		0.57	80		0.22	80		0.092	80		0.33
ECOG (0-1)		—			—			—			—			—	
ECOG (2-3)		-2.4 (-4.9 to 0.06)			0.71 (-1.8 to 3.2)			1.241 (-0.763 to 3.246)			-2.2 (-4.7 to 0.36)			-1.5 (-4.7 to 1.6)	
**PSA**	81	0.00 (-0.01 to 0.00)	0.22	81	0.00 (0.00 to 0.00)	0.30	81	-0.029 (-0.053 to -0.005)	**0.020**	81	-0.03 (-0.06 to 0.00)	0.093	81	-0.01 (-0.05 to 0.03)	0.50
**Gleason Score Category**	78		0.89	78		0.085	78		0.89	78		0.40	78		0.73

Characteristic	Physical			Social			Emotional			Functional			Prostate Cancer Subscale		
	**N**	**Beta (95% CI)**[1]	**p-value**	**N**	**Beta (95% CI)**[1]	**p-value**	**N**	**Beta (95% CI)**[1]	**p-value**	**N**	**Beta (95% CI)**[1]	**p-value**	**N**	**Beta (95% CI)**[1]	**p-value**
6 and 7, Low and Medium Risk		—			—			—			—			—	
8, High Risk		0.12 (-3.4 to 3.6)			-3.2 (-6.6 to 0.25)			0.622 (-2.184 to 3.429)			-1.6 (-5.2 to 1.9)			1.2 (-3.3 to 5.6)	
9 and 10, High Risk		0.68 (-2.5 to 3.9)			-0.09 (-3.2 to 3.0)			0.123 (-2.444 to 2.690)			0.42 (-2.8 to 3.7)			-0.36 (-4.4 to 3.7)	
**Castration Sensitivity**	79		0.10	79		0.99	79		0.94	79		0.48	79		0.24
Castration-Sensitive, CSPC		—			—			—			—			—	
Castration-Resistant, CRPC		-2.1 (-4.6 to 0.41)			0.02 (-2.5 to 2.5)			0.077 (-1.937 to 2.090)			-0.92 (-3.5 to 1.6)			-1.9 (-5.2 to 1.3)	
**Type of ADT Received**	78		0.12	78		0.12	78		0.28	78		0.76	78		0.72
Medical		—			—			—			—			—	
Surgical		-2.2 (-4.9 to 0.58)			2.1 (-0.57 to 4.8)			1.201 (-0.983 to 3.385)			0.44 (-2.4 to 3.3)			-0.65 (-4.2 to 3.0)	
**Burden of Disease**	79		0.78	79		0.16	79		0.62	79		0.27	79		0.41
Low-volume		—			—			—			—			—	
High-volume		-0.36 (-3.0 to 2.3)			-1.8 (-4.4 to 0.72)			-0.520 (-2.592 to 1.551)			-1.5 (-4.1 to 1.2)			-1.4 (-4.8 to 2.0)	
**Number of Treatment Lines Received**	79		0.42	79		0.64	79		0.42	79		0.75	79		0.82
1 Line of Treatment		—			—			—			—			—	
>1 Lines of Treatment		-1.1 (-3.7 to 1.5)			-0.61 (-3.2 to 1.9)			0.827 (-1.213 to 2.867)			-0.42 (-3.0 to 2.2)			-0.39 (-3.7 to 3.0)	

Characteristic	Physical			Social			Emotional			Functional			Prostate Cancer Subscale		
	**N**	**Beta (95% CI)**[1]	**p-value**	**N**	**Beta (95% CI)**[1]	**p-value**	**N**	**Beta (95% CI)**[1]	**p-value**	**N**	**Beta (95% CI)**[1]	**p-value**	**N**	**Beta (95% CI)**[1]	**p-value**
**1st Line Treatment Received**	74		**0.010**	74		0.91	74		0.28	74		0.21	74		0.31
Abiraterone		—			—			—			—			—	
Docetaxel		-4.3 (-7.3 to -1.4)			-0.68 (-3.9 to 2.6)			-1.719 (-4.236 to 0.797)			-3.1 (-6.2 to 0.04)			-2.3 (-6.3 to 1.7)	
Enzalutamide		4.4 (-3.2 to 12)			0.02 (-8.3 to 8.4)			1.745 (-4.759 to 8.249)			-2.7 (-11 to 5.4)			3.8 (-6.4 to 14)	
Fosfosterol		3.9 (-3.7 to 11)			2.4 (-6.0 to 11)			3.745 (-2.759 to 10.25)			2.3 (-5.8 to 10)			6.0 (-4.3 to 16)	
**Duration of 1st Line Treatment Received**	71	0.12 (0.03 to 0.20)	**0.011**	71	-0.02 (-0.12 to 0.07)	0.61	71	0.047 (-0.026 to 0.120)	0.21	71	0.05 (-0.05 to 0.14)	0.31	71	0.12 (0.01 to 0.23)	**0.036**
**Current Treatment Received**	74		0.97	74		0.84	74		0.18	74		0.65	74		**0.015**
ADT+ ARTA		—			—			—			—			—	
ADT + Chemotherapy		-0.05 (-3.1 to 3.0)			0.31 (-2.8 to 3.4)			1.678 (-0.768 to 4.124)			-0.72 (-3.8 to 2.4)			4.7 (0.93 to 8.4)	

[1]CI = Confidence Interval
