# Supplementary material for: Quality of Life Determinants in Patients with Metastatic Prostate Cancer: Insights from a Cross-Sectional Questionnaire-Based Study
Source: Curr Oncol. 2024 Aug 26;31(9):4940–54. doi: 10.3390/curroncol31090366 (PMC11430678; doi:10.3390/curroncol31090366)
Supplement: Supplementary file 1 [file curroncol-31-00366-s001.zip › Supplementary/Table S2.pdf]

Characteristic	Physical		Social		Emotional		Functional		Prostate Cancer Subscale	
	**Beta (95% CI)**[1]	**p-value**	**Beta (95% CI)**[1]	**p-value**	**Beta (95% CI)**[1]	**p-value**	**Beta (95% CI)**[1]	**p-value**	**Beta (95% CI)**[1]	**p-value**
**Age**	-0.01 (-0.18 to 0.16)	0.92	-0.16 (-0.36 to 0.03)	0.10	0.03 (-0.12 to 0.19)	0.68	-0.07 (-0.26 to 0.12)	0.46	-0.14 (-0.35 to 0.08)	0.21
**Number of Comorbidities**		0.10		0.47		0.86		0.64		0.19
None	—		—		—		—		—	
1 Comorbidity	-3.1 (-6.2 to 0.04)		0.73 (-3.0 to 4.4)		0.13 (-2.7 to 3.0)		1.7 (-1.9 to 5.2)		-2.5 (-6.4 to 1.4)	
>1 Comorbidity	-2.9 (-7.3 to 1.4)		3.1 (-2.0 to 8.3)		-1.0 (-5.0 to 3.0)		0.54 (-4.5 to 5.5)		-4.3 (-9.7 to 1.2)	
**Type of Metastasis**		0.66		0.39		0.57		0.21		0.46
Bony metastasis	—		—		—		—		—	
Visceral metastasis	-2.2 (-7.4 to 2.9)		4.1 (-2.0 to 10)		-2.4 (-7.2 to 2.3)		-3.1 (-9.0 to 2.8)		-4.0 (-10 to 2.4)	
Bony and Visceral metastasis	-1.1 (-4.3 to 2.1)		1.8 (-2.0 to 5.6)		-0.44 (-3.4 to 2.5)		1.3 (-2.4 to 4.9)		-0.87 (-4.9 to 3.1)	
**Obese: BMI >25**	2.8 (0.11 to 5.5)	**0.041**	1.2 (-2.0 to 4.3)	0.46	1.8 (-0.68 to 4.2)	0.15	1.5 (-1.6 to 4.5)	0.33	4.4 (1.1 to 7.7)	**0.011**
**ECOG**	-3.3 (-6.2 to -0.51)	**0.022**	0.84 (-2.5 to 4.2)	0.62	0.93 (-1.7 to 3.5)	0.48	-1.6 (-4.8 to 1.7)	0.34	-0.25 (-3.8 to 3.3)	0.89
**PSA**	-0.20 (-0.52 to 0.13)	0.23	0.07 (-0.32 to 0.45)	0.74	-0.35 (-0.65 to -0.05)	**0.024**	-0.25 (-0.62 to 0.12)	0.18	-0.01 (-0.05 to 0.03)	0.58
**Gleason Score Category**		0.74		0.063		0.42		0.62		0.51
6 and 7, Low and Medium Risk	—		—		—		—		—	
8, High Risk	1.5 (-2.4 to 5.3)		-4.6 (-9.2 to -0.07)		2.2 (-1.4 to 5.8)		-2.1 (-6.5 to 2.3)		2.7 (-2.1 to 7.5)	
9 and 10, High Risk	0.87 (-2.4 to 4.1)		-0.27 (-4.1 to 3.6)		0.55 (-2.4 to 3.5)		-0.64 (-4.3 to 3.1)		0.74 (-3.3 to 4.8)	
**Castration Sensitivity**	-0.90 (-3.7 to 1.9)	0.52	-0.14 (-3.4 to 3.1)	0.93	0.72 (-1.8 to 3.3)	0.58	0.21 (-3.0 to 3.4)	0.90	-0.72 (-4.2 to 2.7)	0.68
**1st Line Treatment Received**		0.17		0.61		0.76		0.38		**0.041**

Characteristic	Physical		Social		Emotional		Functional		Prostate Cancer Subscale	
	**Beta (95% CI)[1]**	**p-value**	**Beta (95% CI)[1]**	**p-value**	**Beta (95% CI)[1]**	**p-value**	**Beta (95% CI)[1]**	**p-value**	**Beta (95% CI)[1]**	**p-value**
Abiraterone	—		—		—		—		—	
Docetaxel	-3.6 (-7.0 to -0.10)		-1.8 (-5.9 to 2.2)		-1.7 (-4.9 to 1.5)		-3.3 (-7.3 to 0.64)		-5.9 (-11 to -1.2)	
Enzalutamide	3.7 (-7.9 to 15)		4.8 (-8.9 to 19)		0.03 (-11 to 11)		-3.7 (-17 to 9.6)		-5.2 (-21 to 10)	
Fosfosterol	-2.3 (-11 to 6.7)		3.8 (-6.9 to 14)		1.3 (-7.0 to 9.6)		2.0 (-8.3 to 12)		-11 (-23 to 1.1)	
**Duration of 1st Line Treatment Received**	0.07 (-0.04 to 0.19)	0.19	-0.06 (-0.19 to 0.08)	0.41	0.03 (-0.07 to 0.14)	0.54	0.00 (-0.13 to 0.12)	0.95	0.13 (-0.01 to 0.27)	0.077
**Currently Receiving ADT + ARTA**	-	-	-	-	-	-	-	-	7.9 (3.0 to 13)	**0.002**

[1]CI = Confidence Interval

Table 4: ECOG, Eastern Cooperative Oncology Group; PSA, prostate-specific antigen; BMI, body mass index; ADT, androgen deprivation therapy; ARTA, androgen receptor-targeted agent.
