# Supplementary figures and images for: Quality of Life Determinants in Patients with Metastatic Prostate Cancer: Insights from a Cross-Sectional Questionnaire-Based Study
Source: Curr Oncol. 2024 Aug 26;31(9):4940–54. doi: 10.3390/curroncol31090366 (PMC11430678; doi:10.3390/curroncol31090366)

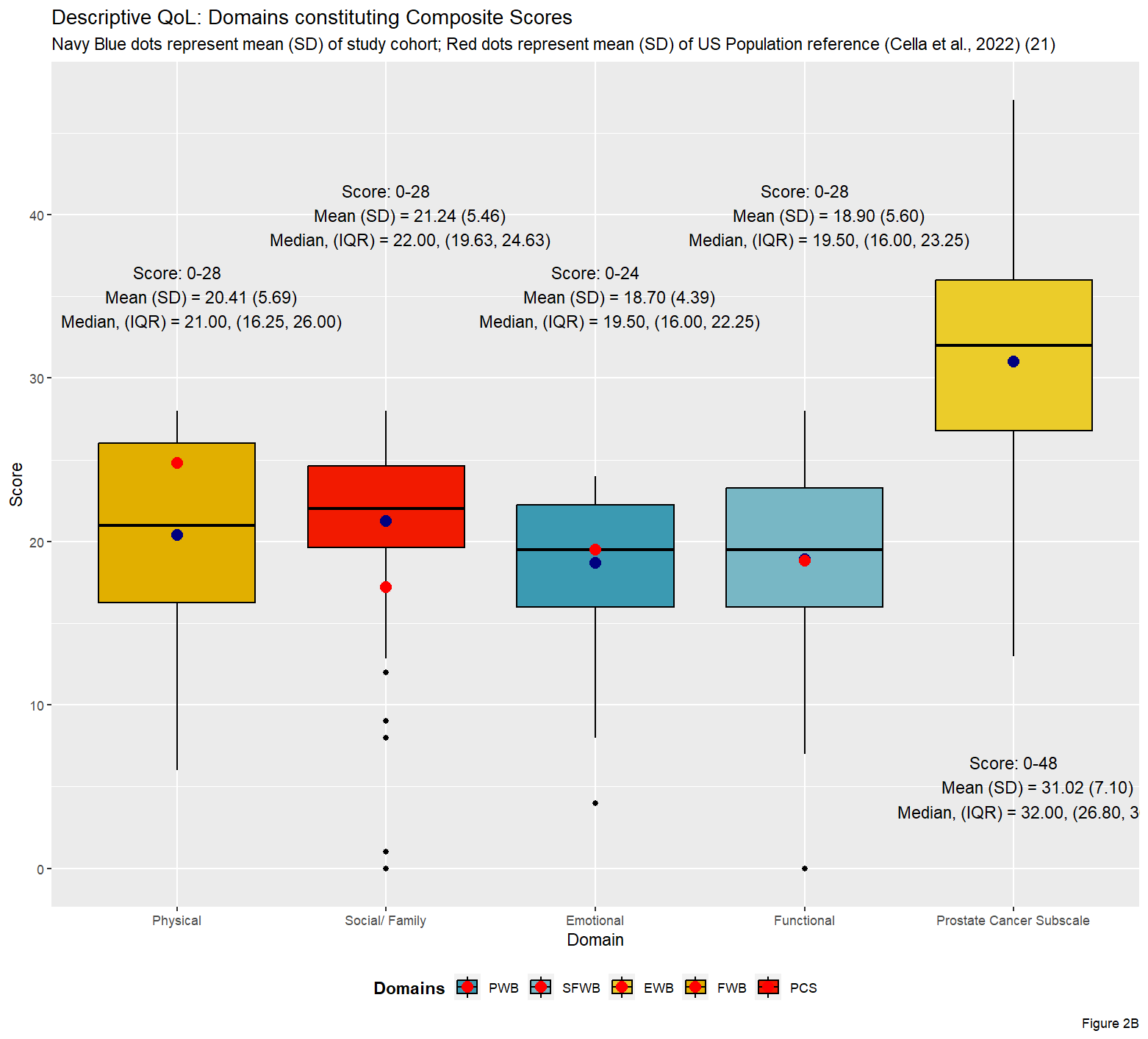

Supplement: Supplementary file 1 [file curroncol-31-00366-s001.zip › Supplementary/Figure S1.png]
